# Carbolic Acid in the Treatment of Conjunctivitis

**Published:** 1869-04

**Authors:** E. L. Holmes


CARBOLIC ACID IN THE TREATMENT OF CONJUNCTIVITIS.—By E. L. HOLMES, M. D., in *American Journal of the Medical Sciences*, October.

Dr. E. L. Holmes states (*Chicago Medical Examiner*) that he has been induced to try the carbolic acid in diseases of the conjunctiva, and he says that he has "found in about a dozen cases, at the infirmary, both of acute, and especially of chronic inflammation, that the patients made a good recovery under its use.

"It is doubtful whether it possesses any advantage over the ordinary astringents, and yet it may be a desirable agent in cases where the usual applications fail. In two cases of purulent conjunctivitis, its use between the applications of nitrate of silver, after the latter had been tried two days, seemed to rapidly overcome the excessive discharge of pus. Possibly, its antiseptic properties may aid in destroying any specific poison that may exist.

"When applied, in a saturated solution, to the conjunctiva, as also to the mucous membrane of the mouth, it produces an intense burning pain, which almost invariably subsides in a few moments. It produces a thin white pellicle on the surface where it is applied, which is soon cast off, leaving scarcely any irritation."

